# Role of cytoreductive nephrectomy in metastatic medullary renal cell carcinoma: controlled analysis

**DOI:** 10.1007/s00345-026-06587-8

**Published:** 2026-09-19

**Authors:** Quynh Chi Le, Andrea Marmiroli, Mattia Longoni, Fabian Falkenbach, Calogero Catanzaro, Michele Nicolazzini, Federico Polverino, Jordan A. Goyal, Fred Saad, Riccardo Schiavina, Luca Fabio Carmignani, Alberto Briganti, Nicola Longo, Markus Graefen, Carlotta Palumbo, Carolin Siech, Severine Banek, Miriam Traumann, Felix K.-H. Chun, Pierre I. Karakiewicz

**Affiliations:** 1 Cancer Prognostics and Health Outcomes Unit, Division of Urology, University of Montréal Health Center, Montréal, Québec Canada; 2 Department of Urology, Goethe University Frankfurt, University Hospital, Frankfurt am Main, Germany; 3 Department of Urology, IEO European Institute of Oncology, IRCCS, Via Ripamonti 435, Milan, Italy; 4 Università degli Studi di Milano, Milan, Italy; 5 Division of Experimental Oncology/Unit of Urology, URI, Urological Research Institute, IRCCS San Raffaele Scientific Institute, Milan, Italy; 6 Vita-Salute San Raffaele University, Milan, Italy; 7 Martini-Klinik Prostate Cancer Center, University Hospital Hamburg- Eppendorf, Hamburg, Germany; 8 Department of Urology, University of Bologna, St. Orsola-Malpighi Hospital, Bologna, Italy; 9 Division of Urology, Department of Translational Medicine, University of Eastern Piedmont, Maggiore della Carità Hospital, Novara, Italy; 10 Division of Urology, Department of Oncology, University of Turin, Orbassano, Italy; 11 Department of Neurosciences, Science of Reproduction and Odontostomatology, University of Naples Federico II, Naples, Italy

**Keywords:** Metastatic renal cell carcinoma, Cytoreductive nephrectomy, Medullary renal cell carcinoma

## Abstract

**Objective:**

To test for differences in overall survival (OS) according to cytoreductive nephrectomy (CN) in medullary metastatic renal cell carcinoma (med-mRCC) relative to most comparable clear cell metastatic RCC (cc-mRCC) patients.

**Methods:**

Within the Surveillance, Epidemiology and End Results (SEER) database (2000–2021), we identified 62 patients with med-mRCC vs. 10,372 with cc-mRCC. Propensity score matching (PSM, ratio 1:3), Kaplan-Meier plots and multivariable Cox regression analyses addressed were used.

**Results:**

Of 10,434 patients, 62 (0.6%) harbored med-mRCC vs. 10,372 (99.4%) cc-mRCC. Med-mRCC patients were younger (27 vs. 63 years, *p* < 0.001), more frequently African American (74 vs. 6%, *p* < 0.001) and more frequently harbored N1 stages (52 vs. 28%, *p* < 0.001). After 1:3 PSM for age, race/ethnicity, T-stage and N-stage, 62 of 62 (100%) med-mRCC (median OS 4 months) and 186 of 10,372 (2%) cc-mRCC patients (median OS 10 months) remained for further analyses. In med-mRCC, median OS was 9 months with CN vs. 3 months without CN (*p* = 0.01). In cc-mRCC, median OS was 28 months with CN vs. 5 months without CN (*p* < 0.001). In med-mRCC, CN independently predicted better OS (Hazard’s Ratio [HR] 0.5, *p* = 0.005), as well as in cc-mRCC (HR 0.4, *p* < 0.001).

**Conclusion:**

Med-mRCC patients exhibit a drastically different phenotype relative to their cc-mRCC counterparts. Despite this difference, med-mRCC exposed to CN harbor a similar relative protective survival effect compared to their cc-mRCC counterparts with most comparable age, race/ethnicity and tumor characteristics.

## Introduction

Renal cell carcinomas (RCC) include a diverse array of histological subtypes, with clear-cell RCC (ccRCC) being the most prevalent subtype, representing approximately 70% of all diagnosed cases [1,2,3]. The remaining RCC cases consist of a heterogenous group of histological variants, collectively referred to as non-ccRCC or variant histology RCC [2]. Among these variants, medullary RCC (medRCC) is one of the most rare and aggressive RCC subtypes [4,5]. MedRCC patients are drastically younger (median age 28), overwhelmingly more frequently of African descent [4,6], usually harbor more advanced stage at initial diagnosis and were associated with poor survival outcomes [4,5,7]. Even when diagnosed at a localized stage, patients with medRCC eventually develop metastases within a few weeks [8,9]. However, medullary metastatic RCC (med-mRCC) has received very little attention in the medical literature, specifically, no previous analyses examined the role of cytoreductive nephrectomy (CN) in med-mRCC yet. We addressed this knowledge gap and tested for differences in overall survival (OS) according to CN vs. no-CN status. Additionally, we compared med-mRCC to clear cell metastatic RCC (cc-mRCC) patients with most comparable age, race/ethnicity and tumor stage characteristics according to CN status. We hypothesized that CN may significantly improve OS in med-mRCC patients. To test this hypothesis, we relied on the Surveillance, Epidemiology and End Results (SEER) database (2000–2021).

## Methods

### Data source and study population

The SEER database provides cancer statistics for approximately 47.9% of the United States population [10]. Within the SEER database (2000–2021), we identified patients aged ≥ 18 with histologically confirmed metastatic RCC (International Classification of Disease for Oncology [ICD-10] site code 64.9) with medRCC (8510/3) or ccRCC (8310/3) subtypes. Moreover, only patients with known SEER stage and survival data were included. Autopsy- or death certificate-only cases were excluded.

### Statistical analyses

**Fig. 1:**
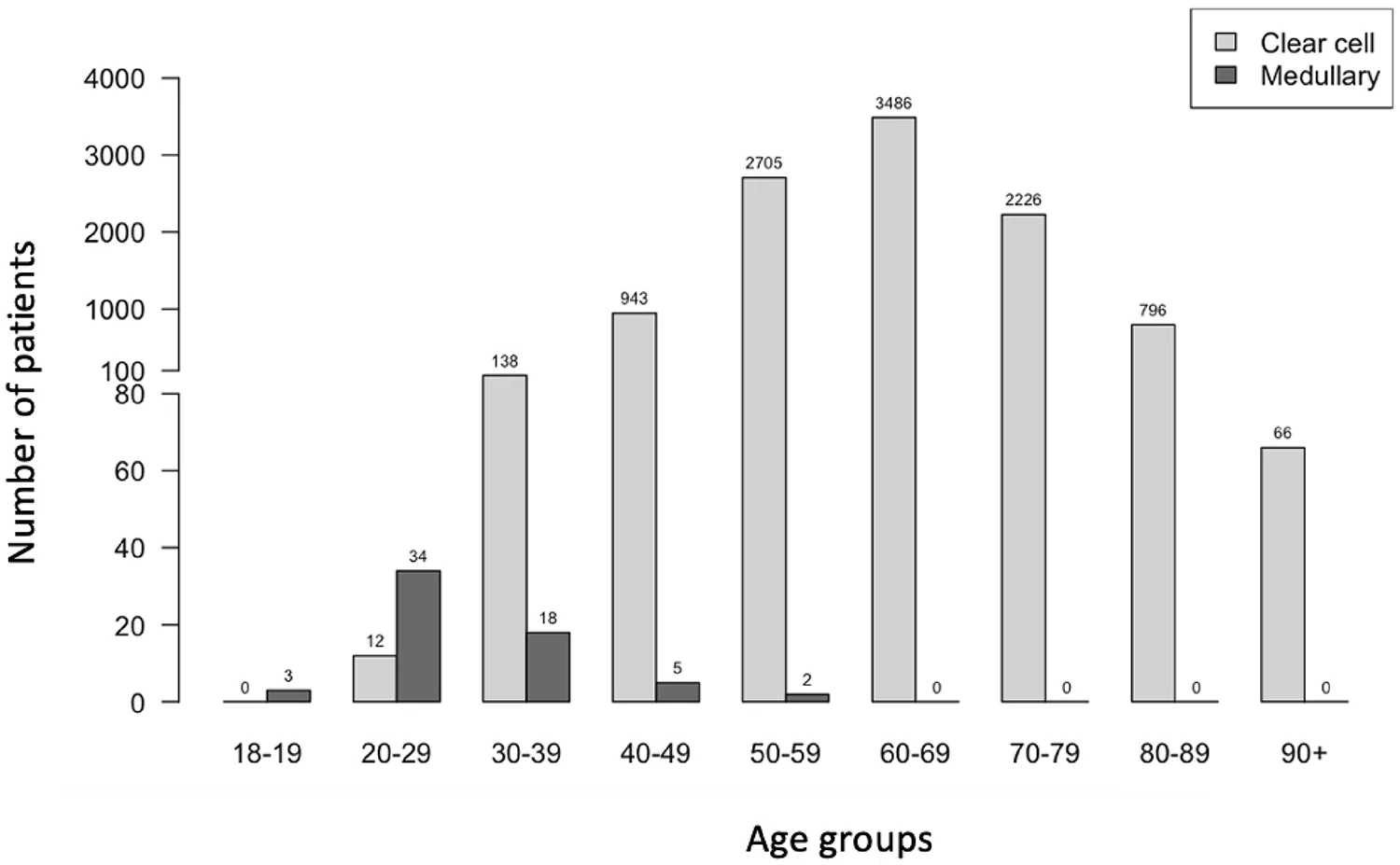
Barplots depicting distribution of age in metastatic medullary RCC and clear cell RCC (SEER 2000–2021)

Baseline characteristics were tabulated (Tables 1 and 2). For continuous variables, medians and ranges were included, while frequencies and proportions were reported for categorical variables. Second, propensity-score matching (PSM) was applied (ratio 1:3) for age, race/ethnicity, T-stage and N-stage. Due to major differences in age between med-mRCC and cc-mRCC, matching was only possible for age-groups (< 29 years and by each decade between 30 and 90 years). Third, Kaplan-Meier plots addressed OS in med-mRCC and cc-mRCC and in subgroups according to CN status (Fig. 1). Univariable and multivariable Cox regression analyses addressed the association of CN status and OS (Table 3). Statistical tests were two-sided with a level of significance set at *p* < 0.05. R software environment (R Version 4.4.0, The R Foundation for Statistical Computing, Vienna, Austria) was applied for graphics and statistical computing.

**Table 1:** Descriptive characteristics of 10,434 metastatic medullary and clear cell RCC in the SEER database 2000–2021 according to histological subtype and receipt of cytoreductive nephrectomy, before and after 1:3 propensity score matching

Characteristic	Before PSM				After 1:3 PSM			
	Overall *n* = 10,434	Medullary RCC *n* = 62 (0.6%)^1^	Clear cell RCC *n* = 10,372 (99.4%)1	*p*-value	Overall *n* = 248	Medullary *n* = 62 (25%)^1^	Clear cell RCC *n* = 186 (75%)^1^	*p*-value
Age	63 (56, 71)	27 (22, 35)	63 (56, 71)	< 0.001	50 (38, 55)	27 (22, 35)	53 (47, 57)	< 0.001
Male sex	7149 (69%)	45 (73%)	7104 (68%)	0.5	174 (70%)	45 (73%)	129 (69%)	0.6
T Stage				0.3				1.0
T1-2	2087 (20%)	16 (26%)	2071 (20%)		64 (26%)	16 (26%)	48 (26%)	
T3-4	8347 (80%)	46 (74%)	8301 (80%)		184 (74%)	46 (74%)	138 (74%)	
N Stage				< 0.001				1.0
N0/Nx	7474 (72%)	30 (48%)	7444 (72%)		120 (48%)	30 (48%)	90 (48%)	
N1	2960 (28%)	32 (52%)	2928 (28%)		128 (52%)	32 (52%)	96 (52%)	
Race				< 0.001				1.0
Caucasian	7305 (70%)	3 (5%)	7302 (70%)		12 (5%)	3 (5%)	9 (5%)	
African American	669 (6%)	46 (74%)	623 (6%)		184 (74%)	46 (74%)	138 (74%)	
Other	2460 (24%)	13 (21%)	2447 (24%)		52 (21%)	13 (21%)	39 (21%)	
CN				0.2				0.6
Yes	5939 (57%)	30 (48%)	5909 (57%)		127 (51%)	30 (48%)	97 (52%)	

Abbreviations:*PSM*propensity score matching,*CN* cytoreductive nephrectomy, RCC renal cell carcinoma

^1^Median (Q1, Q3);*n*(%)

**Table 2:** Descriptive characteristics of 62 metastatic renal medullary and 186 clear cell RCC in the SEER database 2000–2021 stratified according to receipt of cytoreductive nephrectomy

Characteristic	Medullary RCC *n* = 62 (25%)			Clear cell RCC *n* = 186 (75%)		
	Cytoreductive nephrectomy *n* = 30 (48%)^1^	No cytoreductive nephrectomy *n* = 32 (52%)^1^	*p*-value	Cytoreductive nephrectomy *n* = 97 (52%)^1^	No cytoreductive nephrectomy *n* = 89 (48%)^1^	*p*-value
Age	30 (20, 56)	24 (18, 39)	0.01	53 (48, 56)	53 (47, 57)	0.9
Male sex	24 (80%)	21 (66%)	0.2	67 (69%)	62 (70%)	0.9
T Stage			0.3			0.10
T1-2	6 (20%)	10 (31%)		30 (31%)	18 (20%)	
T3-4	24 (80%)	22 (69%)		67 (69%)	71 (80%)	
N Stage			0.8			1.0
N0/Nx	14 (47%)	16 (50%)		47 (48%)	43 (48%)	
N1	16 (53%)	16 (50%)		50 (52%)	46 (52%)	
Race			0.2			0.4
Caucasian	3 (10%)	0 (0%)		5 (5%)	4 (5%)	
African Americans	22 (73%)	24 (75%)		68 (70%)	70 (79%)	
Other	5 (17%)	8 (25%)		24 (25%)	15 (17%)	

^1^Median (Q1, Q3);*n*(%)

**Table 3:** Multivariable Cox regression models addressing overall survival in (a) metastatic medullary RCC and (b) metastatic clear cell RCC (SEER 2000–2021) according to receipt of cytoreductive nephrectomy

Characteristic		Univariable				Multivariable*		
		HR^1^	95% CI^1^	*p*-value		HR^1^	95% CI^1^	*p*-value
*(a) Metastatic medullary RCC*								
CN (Ref: no CN)								
CN performed		0.5	0.3, 0.9	**0.016**		0.5	0.3, 0.9	**0.019**
*(b) Metastatic ccRCC*								
CN (Ref: no CN)								
CN performed		0.4	0.3, 0.6	**< 0.001**		0.4	0.3, 0.5	**< 0.001**

^1^*HR* Hazard Ratio, *CI*  Confidence Interval, *CN* cytoreductive nephrectomy

*Adjusted for age, sex, race

Bold values are the p-values, they are highlighted due to statistically significance.

## Results

### Descriptive characteristics of med-mRCC and cc-mRCC patients

**Fig. 2:**
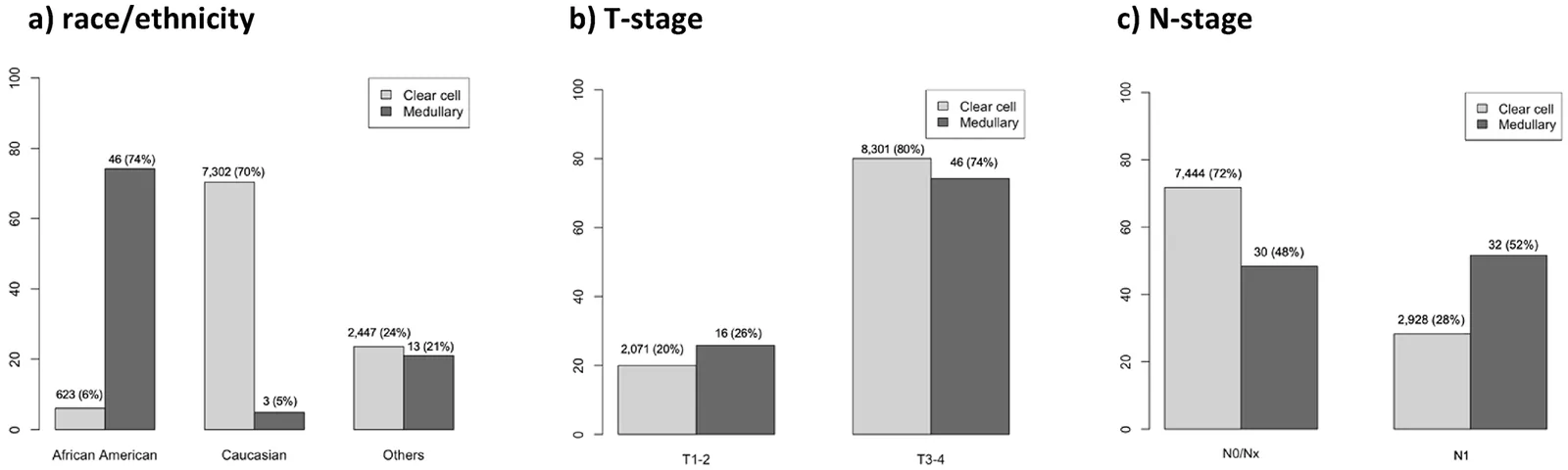
Barplots depicting distribution of (**a)** race/ethnicity and (**b)** T-stages and (**c)** N-stages in metastatic medullary RCC and clear cell RCC (SEER 2000–2021)

**Fig. 3:**
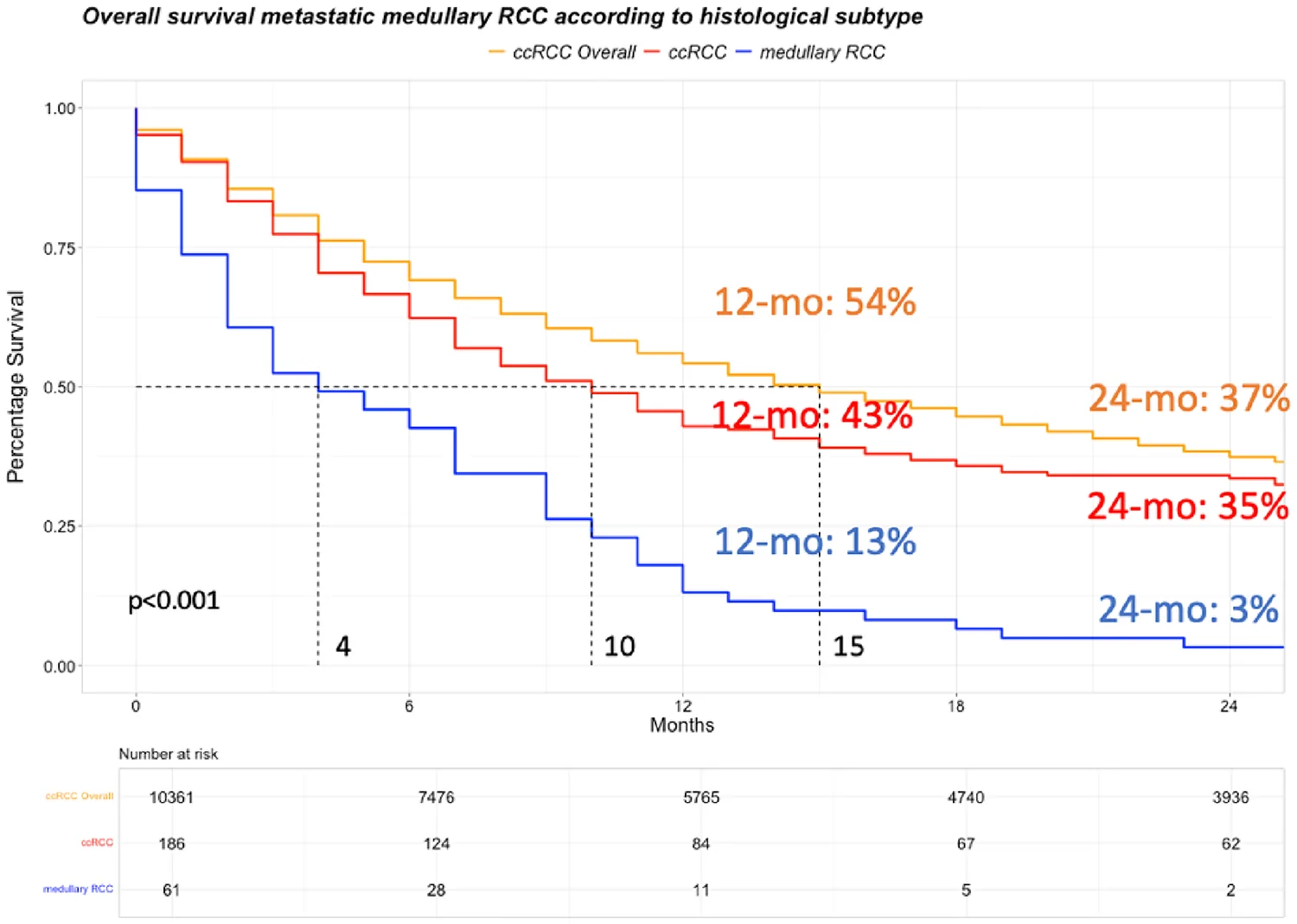
Kaplan Meier curves depicting overall survival of metastatic medullary RCC and clear cell RCC in the overall cohort (SEER 2000–2021)

Within the SEER database (2000–2021), we identified 10,434 metastatic RCC patients. Of those, 62 (0.6%) harbored med-mRCC and 10,372 (99.4%) cc-mRCC (Table 1). Med-mRCC patients were younger (27 vs. 63 years, *p* < 0.001), more frequently African American (74 vs. 6%, *p* < 0.001) and more frequently harbored N1 stages (52 vs. 28%, *p* < 0.001). Regarding age, all med-mRCC patients were aged < 56 years. Conversely, only a third of cc-mRCC (36%) were < 60 years (Fig. 1). Regarding race/ethnicity, med-mRCC displayed a different distribution compared to cc-mRCC: 74% of med-mRCC were African American vs. 6% Caucasian vs. 21% other race/ethnicity (Fig. 2). Conversely, 70% of cc-mRCC patients were Caucasian vs. 6% African American vs. 24% other race/ethnicity. The above differences were addressed in a PSM for age, race/ethnicity, T-stage and N-stage for the purpose of subsequent comparison between med-mRCC and cc-mRCC. After 1:3 PSM, 62 of 62 (100%) med-mRCC and 186 of 10,372 (2%) cc-mRCC patients remained for further analyses. The only significant difference between med-mRCC vs. cc-mRCC after PSM was age (27 vs. 53 years, *p* < 0.001) (Fig. 3).

### CN in med-mRCC and cc-mRCC patients

Regarding rates of CN, 30 of 62 (48%) med-mRCC and 97 of 186 (52%) cc-mRCC patients underwent CN (Table 2). Med-mRCC patients with CN were significantly older compared to their med-mRCC counterparts without CN (30 vs. 24 years, *p* = 0.01). No further differences were observed between CN and non-CN med-mRCC patients. Similarly, in the matched cc-mRCC cohort, no differences were observed between CN and non-CN cc-mRCC patients.

### OS differences in med-mRCC vs. cc-mRCC

OS was examined in med-mRCC patients relative to most comparable cc-mRCC patients as well as to all cc-mRCC patients. In 62 med-mRCC, median OS was 4 months. Conversely, median OS was 10 months in 186 matched cc-mRCC and 15 months in 10,361 cc-mRCC patients (*p* < 0.001). OS-rates were 13 vs. 43 vs. 54% at 12 months and 3 vs. 35 vs. 37% at 24 months, respectively (Fig. 1).

### OS according to CN status in med-mRCC

**Fig. 4:**
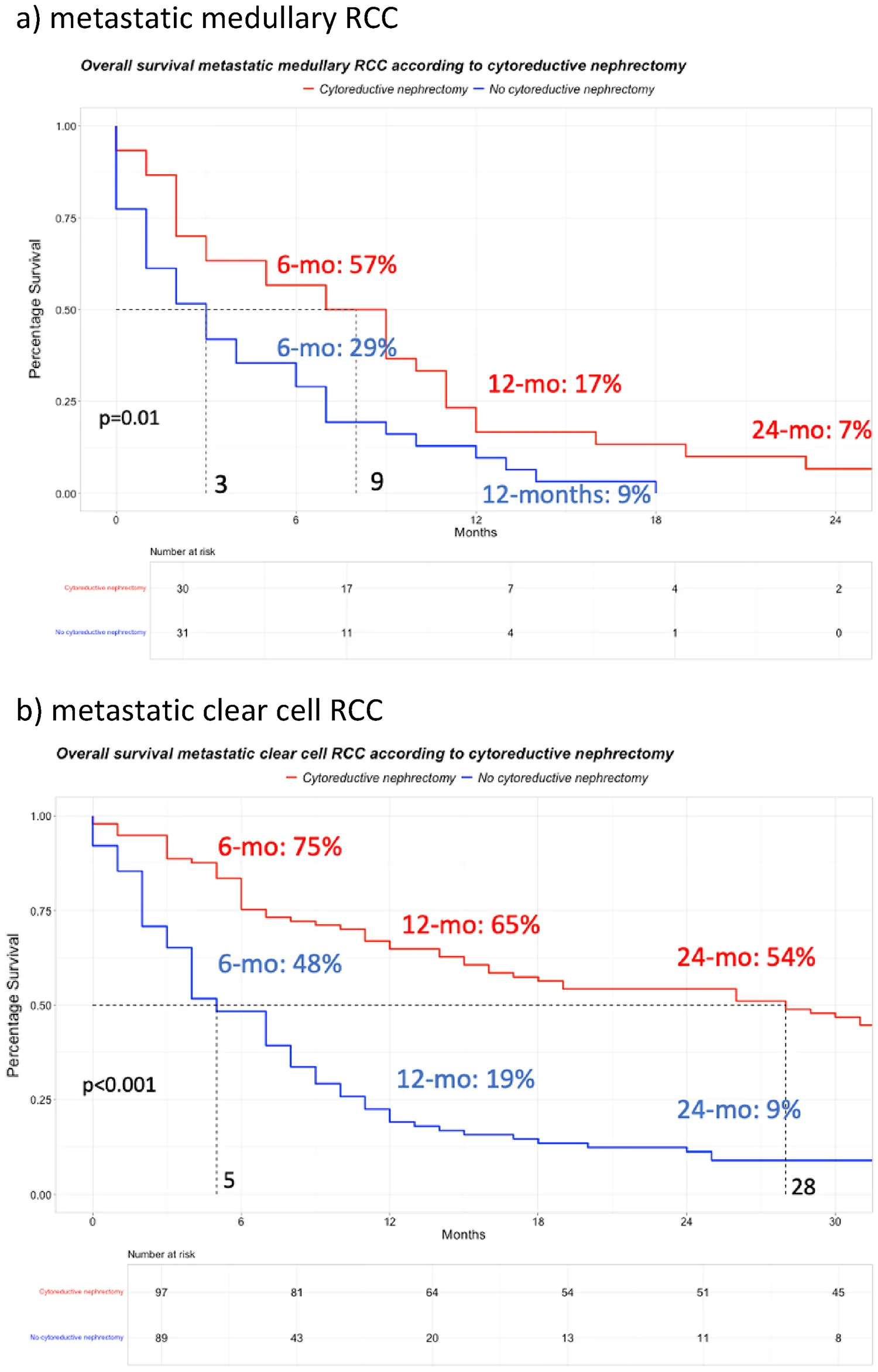
Kaplan Meier curves depicting overall survival of (**a)** metastatic medullary RCC and (**b)** metastatic clear cell RCC according to cytoreductive nephrectomy status (SEER 2000–2021)

Of 62 patients with med-mRCC, 30 (48%) underwent CN (Fig. 4A). Median OS was 9 vs. 3 months in med-mRCC patients with CN vs. without CN. At 6 months of follow-up, OS rates were 57 vs. 29% in med-mRCC patients with CN vs. without CN, respectively. At 12 months of follow-up, OS rates were 17 vs. 9% in med-mRCC patients with CN vs. without CN, respectively. In univariable Cox regression models (Table 3A), CN was associated with improved OS(HR 0.5, p = 0.016). After adjustment for age, race/ethnicity and sex, CN reached independent predictor status for OS (HR 0.5, p = 0.019). 

### OS according to CN status in cc-mRCC

Of 186 patients with cc-mRCC, 97 (52%) underwent CN (Fig. 4B). Median OS was 28 months vs. 5 months in cc-mRCC patients with CN vs. without CN. At 6 months of follow-up, OS rates were 75 vs. 48% in cc-mRCC patients with CN vs. without CN, respectively. At 12 months of follow-up, OS rates were 65 vs. 19% in cc-mRCC patients with CN vs. without CN, respectively. In univariable Cox regression models (Table 3), CN was associated with improved OS (HR 0.4, *p* < 0.001). After adjustment for age, race/ethnicity and sex, CN reached independent predictor status for OS (HR 0.4, *p* < 0.001).

## Discussion

Currently, the role of CN in med-mRCC is unknown. We examined the association between CN and OS in med-mRCC patients. Additionally, we compared the med-mRCC observations with those of most similar cc-mRCC patients. We hypothesized that CN may be associated with improved OS in med-mRCC patients and made several noteworthy observations.

First, within the SEER database (2000–2021) only 62 med-mRCC were identified. Conversely, 10,372 cc-mRCC patients were identified within the same study period. These observations underline the rarity of medRCC [4,5,9,11]. In consequence, studies addressing medRCC are scarce, particularly with focus on metastatic stage. This explains why no previous studies examined the association of CN within med-mRCC patients. Absence of such analysis validate the need for the current study.

Second, baseline characteristics in med-mRCC patients were drastically different than those of their cc-mRCC counterparts. Specifically, med-mRCC patients were considerably younger (median age 27 vs. 63 years, *p* < 0.001) and predominantly African American (74%). Only a very small proportion of Caucasian harbor med-mRCC (5%). Conversely, cc-mRCC were predominantly Caucasian (70%) and only a small proportion of African American (6%) harbored cc-mRCC. Finally, med-mRCC exhibited a higher proportion of N1 stage at diagnosis relative to their cc-mRCC counterparts (52 vs. 28%, *p* < 0.001). Presence of age and race/ethnicity differences is substantially noteworthy and, without doubt, contribute to profoundly different outcomes of med-mRCC relative to cc-mRCC. Due to presence of these differences, adjustment was required and PSM was applied. For purpose of contrasting, survival outcomes of med-mRCC patients were compared to their most similar cc-mRCC counterparts.

Third, OS of med-mRCC patients (*n* = 62) relative to their most comparable cc-mRCC counterparts (*n* = 186) and all cc-mRCC counterparts (*n* = 10,361) exhibited very important differences. Specifically, med-mRCC exhibited median OS of 4 months vs. 10 months in matched cc-mRCC vs. 15 months in all cc-mRCC. These differences validate a drastically more aggressive phenotype of med-mRCC relative to that of even most closely matched cc-mRCC patients. Interestingly, after matching for age, race/ethnicity and tumor stage, the matched cc-mRCC cohort exhibited significantly less favorable survival than the cc-mRCC overall cohort. It is noteworthy that the med-mRCC cohort OS of 4 months is superior to the survival-cutoff of < 3 months as recommended by Heng et al. for consideration of CN [12]. In consequence, based on OS, med-mRCC patients may be considered for CN according to those well-established CN selection criteria. Based on this consideration, further analyses addressing the association between CN and OS were justified.

Fourth, the association between CN status and OS was studied in both, med-mRCC as well as in their most comparable cc-mRCC counterparts. In med-mRCC patients, median OS was 9 vs. 3 months in patients with vs. without CN, respectively. After multivariable adjustment, this resulted in a HR 0.5 (*p* = 0.019). The same analyses in matched cc-mRCC patients yielded median OS of 28 vs. 5 months for CN vs. no CN, respectively and a HR 0.4 (*p* < 0.001). These observations indicate a very strong association between CN and longer survival in med-mRCC. Moreover, this association is extremely comparable to the one recorded in most comparable cc-mRCC patients treated with either CN or no CN. In med-mRCC patients, 9 vs. 3 months median OS difference may be interpreted as a criterion for consideration of CN in those individuals. Despite this sharp difference in median OS, very few med-mRCC patients remain alive past 24 months of follow-up. Specifically, only 7% of such patients were identified in the current analysis even when CN was performed vs. 0% when no CN was performed.

Taken together, med-mRCC patients are very different from their cc-mRCC counterparts. Age and race/ethnicity contribute to most striking differences and clearly underline a different phenotype that is associated with substantially worse survival. Even when these differences are accounted for and cc-mRCC patients with most similar characteristics to their med-mRCC counterparts are selected, a strong survival disadvantage still persists in med-mRCC patients. The association between CN and OS in med-mRCC is very similar to that of CN in cc-mRCC patients as evidenced by virtually the same HR (respective HR 0.5 and HR 0.4, both *p* < 0.05). Additionally, med-mRCC patients have very important OS difference in absolute value that is evidenced by 9 months median OS after CN relative to only 3 months when CN is not performed. These values sharply contrast with median OS recorded in most comparable cc-mRCC counterparts with median OS of 28 vs. 5 months. The above observations validate the presence of a survival benefit signal associated with CN in med-mRCC. In consequence, such option should be systematically considered in treatment decision making within those individuals. However, while CN could improve OS and extend survival for several months, the decision to pursue or forgo CN should be made carefully. Considering potential adverse effects of CN, including postoperative complications and in-hospital, CN may not be suitable for all med-mRCC patients.

Despite its novelty, this study has limitations due to its observational design and the retrospective nature of the SEER database. First and foremost, despite the large scale of the SEER database, the small sample size of metastatic med-mRCC patients is limited due to low numbers of observations, since med-mRCC is a very rare malignancy. Second, we could not assess further comorbidities and laboratory results and the use of established prognostic risk factors in med-mRCC, such as IMDC (International metastatic RCC database consortium) criteria in our survival analyses, since this detailed information is not provided within the SEER database. Third, detailed information regarding systemic therapies, including timing, type and sequence of treatment was not avaiable in the SEER database, particularly for cc-mRCC patients. Consequently, differences attributable to the the administration of systemic therapy could not be accounted for in the current analyses. Lastly, we were unable to address cancer-specific-mortality and other-cause mortality in med-mRCC patients due to the limited and relatively small cohort of only 62 patients. However, given the rarity of med-mRCC, it is unlikely that any other study will have significantly larger sample sizes. Thus, no more detailed information on survival is expected to be made.

## Conclusion

Med-mRCC patients harbor drastically different baseline characteristics and survival outcomes after CN compared to their cc-mRCC counterparts. Med-mRCC patients were younger, more frequently African American and more frequently harbored advanced N-stages. However, CN reached independent predictor status for improved OS in both, med-mRCC and cc-mRCC patients, suggesting potential benefit derived from CN for these patient cohorts.

## Data Availability

No datasets were generated or analysed during the current study.
